# Comparison of Urinary Isolate Distribution and Antimicrobial Resistance Profiles in Kidney Transplant Recipients and Non-Transplant Nephrology Patients

**DOI:** 10.3390/antibiotics15080720

**Published:** 2026-07-24

**Authors:** Büşra Çalışır, Abdullah İbrahim Çalışır, Safa Şanda, Nazmiye Ülkü Tüzemen, Abdülmecit Yıldız, Alparslan Ersoy, Cüneyt Özakın

**Affiliations:** 1 Department of Medical Microbiology, Bursa Uludağ University, 16059 Bursa, Türkiye; safasanda@uludag.edu.tr (S.Ş.); utuzemen@uludag.edu.tr (N.Ü.T.); ozakin@uludag.edu.tr (C.Ö.); 2 Bursa Gemlik State Hospital, 16600 Bursa, Türkiye; calisiribrahim@gmail.com; 3 Division of Nephrology, Department of Internal Medicine, Bursa Uludağ University, 16059 Bursa, Türkiye; mecit@uludag.edu.tr (A.Y.); alpersoy@uludag.edu.tr (A.E.)

**Keywords:** urinary tract infection, urine culture, urinary isolates, kidney transplantation, nephrology, antimicrobial resistance, antimicrobial resistance surveillance

## Abstract

**Background/Objectives:** Urinary tract infections (UTIs) are common infectious complications in kidney transplant recipients (KTRs) and non-transplant nephrology patients. However, direct culture-based comparisons of urinary isolate distribution and antimicrobial resistance profiles between KTRs and non-transplant nephrology (NTN) patients remain limited. This study compared urinary isolates recovered from urine cultures with significant growth and their in vitro antimicrobial resistance profiles in hospitalized KTRs and NTN patients. **Methods:** In this retrospective culture-based study, urine culture results from patients hospitalized in the nephrology and kidney transplantation units between 1 January 2024 and 31 December 2025 were reviewed. Urinary isolate distribution, in vitro antimicrobial resistance profiles, and extended-spectrum β-lactamase (ESBL) production were compared between the two. **Results:** A total of 397 patients with at least one urine culture showing significant growth were included, comprising 220 KTRs and 177 NTN patients. Enterobacterales were significantly more frequent among KTRs than among NTN patients (77.2% vs. 41.1%, *p* < 0.001). *Escherichia coli* was the predominant urinary isolate in both groups. In contrast, *Candida* spp. (26.8% vs. 3.2%, *p* < 0.001), *Enterococcus* spp. (15.8% vs. 7.7%, *p* < 0.001), and non-fermenting Gram-negative bacteria (9.4% vs. 3.4%, *p* = 0.001) were more frequently recovered from NTN patients. Among *E. coli* isolates, resistance to gentamicin (*p* = 0.006) and ertapenem (*p* = 0.001) was significantly higher in the NTN group. Likewise, *Klebsiella pneumoniae* isolates from NTN patients showed significantly higher resistance to third-generation cephalosporins, aminoglycosides, and carbapenems (*p* < 0.05). ESBL production was also more frequent in NTN patients than in KTRs (55.7% vs. 41.9%, *p* = 0.013). **Conclusions:** Hospitalized KTRs and NTN patients exhibited distinct urinary isolate distributions and in vitro antimicrobial resistance profiles. These findings should be interpreted as culture-based epidemiological data rather than as differences in clinically confirmed UTI burden. Stratified local surveillance may help contextualize urinary isolate and susceptibility patterns across hospitalized nephrology populations.

## 1. Introduction

Urinary tract infections (UTIs) are common infectious complications in kidney transplant recipients (KTRs) and patients hospitalized in nephrology units, contributing substantially to clinical burden [1,2]. In KTRs, UTIs, particularly those occurring during the early post-transplant period, have been associated with impaired graft function, acute rejection, and sepsis [3,4,5]. However, positive urine cultures do not necessarily indicate clinically symptomatic UTI. In retrospective laboratory-based studies, urine cultures may reflect symptomatic infection, asymptomatic bacteriuria, candiduria, colonization, or contamination, particularly when clinical symptoms, pyuria, urine culture indication, and catheter status are not systematically available [4,6,7].

The increasing prevalence of extended-spectrum β-lactamase (ESBL)-producing organisms and multidrug resistance (MDR) complicates the interpretation of urinary isolate susceptibility patterns and may affect empirical antimicrobial decision-making when treatment is clinically indicated [1,8,9,10]. Accordingly, ongoing surveillance of local urinary isolate distributions and antimicrobial susceptibility patterns is important [11,12]. KTRs and non-transplant nephrology (NTN) patients may differ with respect to immunosuppression, urinary tract instrumentation, prophylactic antimicrobial exposure, kidney function, and healthcare exposure. These characteristics may be associated with variation in urinary isolate distribution and antimicrobial resistance profiles between the two populations [1,2,3,4].

In a previous single-center study at our institution that included only KTRs, Gram-negative urinary isolates predominated, resistance to ciprofloxacin and trimethoprim–sulfamethoxazole (TMP-SMX) was high, and carbapenem resistance was relatively low [13]. However, that study focused exclusively on KTRs and did not include a non-transplant nephrology comparator group. Whether these patterns differ from those observed in NTN patients managed within the same institutional setting during the same study period therefore remains unclear. Although microbiological data have been reported separately for both populations, direct culture-based comparisons of urinary isolate distribution and antimicrobial resistance profiles between hospitalized KTRs and NTN patients remain limited [14,15]. The present study addresses this gap by directly comparing contemporaneous urine culture data from KTRs and NTN patients managed at the same tertiary-care institution during the same two-year study period. This culture-based comparative approach may help contextualize urinary isolate distribution and antimicrobial resistance profiles across two distinct hospitalized nephrology populations while minimizing differences related to laboratory methods and study period. Therefore, this study aimed to compare the distribution of urinary isolates recovered from urine cultures with significant growth and their in vitro antimicrobial resistance profiles between hospitalized KTRs and NTN patients at a tertiary-care university hospital.

## 2. Results

### 2.1. Demographic Information of the Study Group

Of the 1638 urine cultures evaluated, 1079 were obtained from kidney transplant recipients (KTRs) and 559 from non-transplant nephrology (NTN) patients. The proportion of cultures with significant growth was 470/1079 (43.6%) among KTRs and 265/559 (47.4%) among NTN patients, with no statistically significant difference between groups (*p* = 0.147). In contrast, contamination was more frequent among cultures from KTRs than among those from NTN patients (173/1079, 16.0% vs. 25/559, 4.5%; *p* < 0.001) (Table 1).

Patient-level characteristics of the 397 patients with at least one culture showing significant growth are summarized in Table 1 (220 KTRs and 177 NTN patients). The mean number of cultures with significant growth per patient was higher in KTRs than in NTN patients (2.14 vs. 1.50; *p* < 0.001). KTRs were younger than NTN patients, with median ages of 49.5 years (range, 21–83) and 72.0 years (range, 20–98), respectively (*p* < 0.001). Female sex was more frequent among KTRs than among NTN patients (173/220, 78.6% vs. 104/177, 58.7%; *p* < 0.001).

### 2.2. Distribution of Urinary Isolates

**Figure 1:**
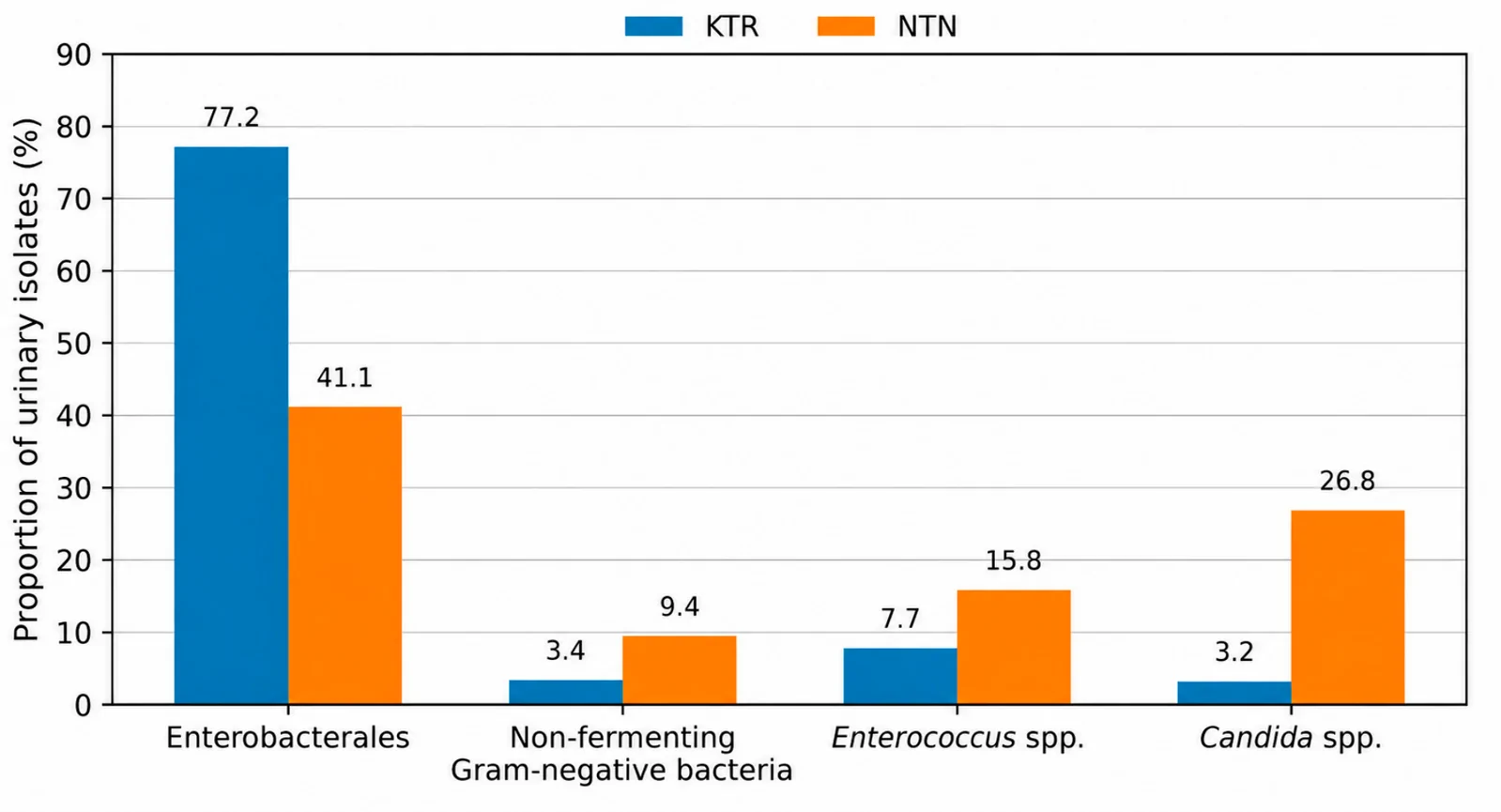
Distribution of major urinary isolate groups among kidney transplant recipients (KTRs) and non-transplant nephrology (NTN) patients. Percentages were calculated within each group of urinary isolates. KTRs, kidney transplant recipients; NTN, non-transplant nephrology patients.

The distribution of urinary isolates by patient group and taxon is shown in Table 2 and Figure 1. Enterobacterales were the predominant group of isolates in both cohorts, and *Escherichia coli* was the most frequently isolated species. However, the distribution of major microbial groups differed between KTRs and NTN patients. Enterobacterales were more frequently isolated from KTRs than from NTN patients (363/470, 77.2% vs. 109/265, 41.1%; *p* < 0.001), including *E. coli* (248/470, 52.8% vs. 64/265, 24.2%; *p* < 0.001) and *Klebsiella pneumoniae* (95/470, 20.2% vs. 32/265, 12.1%; *p* = 0.007). In contrast, non-fermenting Gram-negative bacteria were more frequently isolated from NTN patients than from KTRs (25/265, 9.4% vs. 16/470, 3.4%; *p* = 0.001). *Enterococcus* spp. were also more frequently isolated from NTN patients (42/265, 15.8% vs. 36/470, 7.7%; *p* < 0.001), particularly *Enterococcus faecium* (17/265, 6.4% vs. 3/470, 0.6%; *p* < 0.001). *Candida* spp. were also more frequently isolated from NTN patients than from KTRs (71/265, 26.8% vs. 15/470, 3.2%; *p* < 0.001).

### 2.3. Antimicrobial Resistance Profiles

#### 2.3.1. Resistance Profiles of Gram-Negative Urinary Isolates

**Figure 2:**
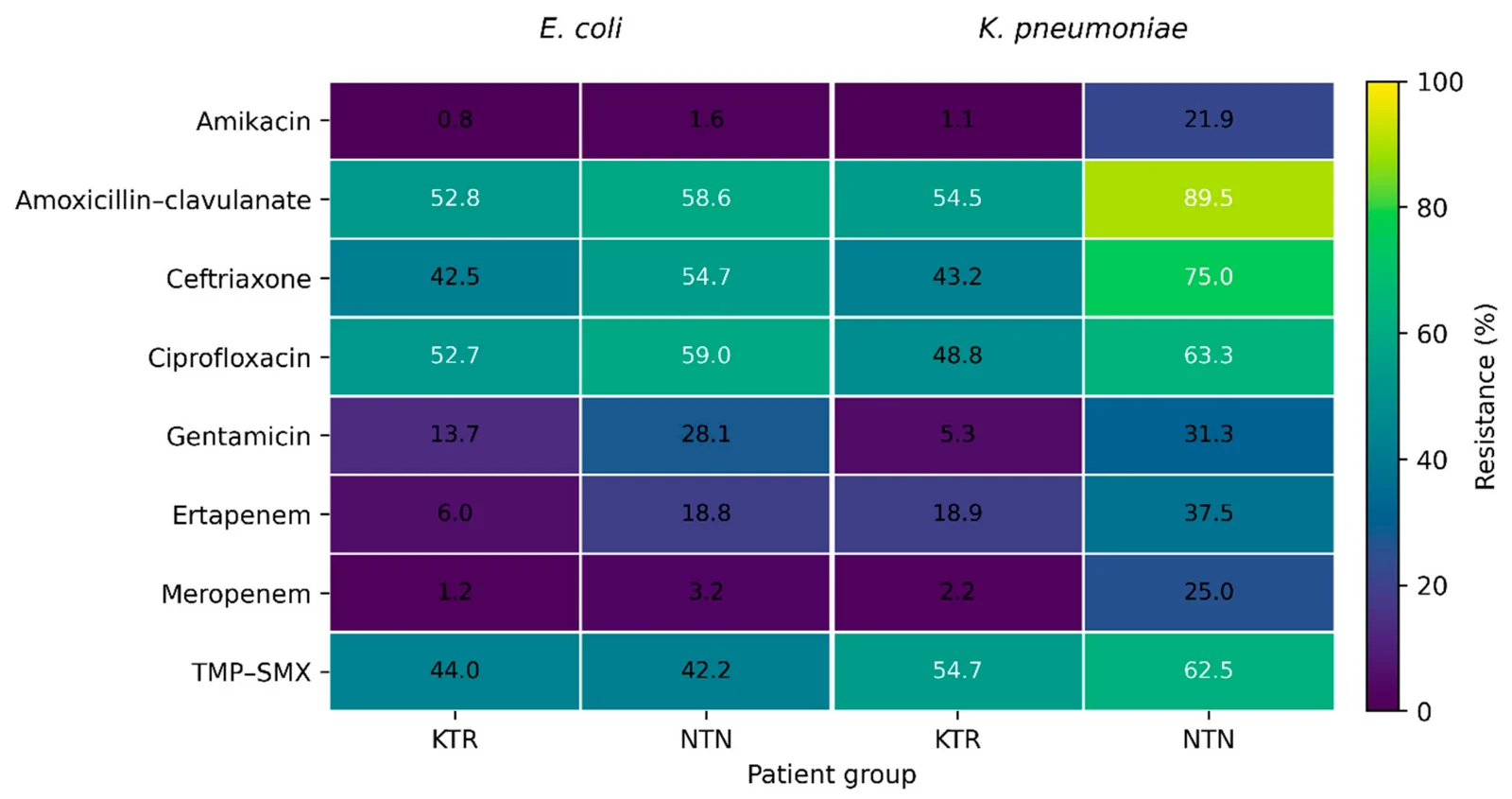
Heatmap of antimicrobial resistance rates among *Escherichia coli* and *Klebsiella pneumoniae* urinary isolates from KTRs and NTN patients. Cell values represent the percentage of resistant isolates among isolates tested for each antimicrobial agent. Denominators are reported in Table 3. KTR, kidney transplant recipients; NTN, non-transplant nephrology patients; TMP–SMX, trimethoprim–sulfamethoxazole.

The antimicrobial resistance profiles of *E. coli* and *K. pneumoniae* isolates are summarized in Table 3 and Figure 2. These species were the most frequently isolated Gram-negative bacteria in the study. Among *E. coli* isolates, resistance to gentamicin was higher among isolates from NTN patients than among those from KTRs (18/64, 28.1% vs. 34/248, 13.7%; *p* = 0.006). Ertapenem resistance was also higher among NTN isolates (12/64, 18.8% vs. 15/248, 6.0%; *p* = 0.001). Among *K. pneumoniae* isolates, resistance was higher in NTN patients than in KTRs for amoxicillin–clavulanate (17/19, 89.5% vs. 30/55, 54.5%; *p* = 0.006), ceftriaxone (24/32, 75.0% vs. 41/95, 43.2%; *p* = 0.002), amikacin (7/32, 21.9% vs. 1/95, 1.1%; *p* < 0.001), gentamicin (10/32, 31.3% vs. 5/95, 5.3%; *p* < 0.001), ertapenem (12/32, 37.5% vs. 18/95, 18.9%; *p* = 0.033), and meropenem (8/32, 25.0% vs. 2/93, 2.2%; *p* < 0.001). Amikacin showed the lowest resistance rates for both *E. coli* and *K. pneumoniae* isolates in both patient populations. Among Enterobacterales isolates tested for extended-spectrum β-lactamase (ESBL) production, ESBL positivity was more frequent among NTN patients than among KTRs (59/106, 55.7% vs. 148/353, 41.9%; *p* = 0.013).

Owing to the limited number of non-fermenting Gram-negative isolates, no formal statistical comparisons were performed; therefore, these findings should be interpreted descriptively and cautiously. Among *Pseudomonas aeruginosa* isolates, imipenem resistance was observed in 0/2 KTR isolates (0.0%) and 4/5 NTN isolates (80.0%), while meropenem resistance was observed in 0/2 KTR isolates (0.0%) and 2/4 NTN isolates (50.0%). Ceftazidime resistance was observed in 0/2 KTR isolates (0.0%) and 3/5 NTN isolates (60.0%). Ciprofloxacin resistance was observed in 1/2 KTR isolates (50.0%) and 2/5 NTN isolates (40.0%), whereas amikacin resistance was observed in 0/2 KTR isolates (0.0%) and 2/5 NTN isolates (40.0%). Among *Acinetobacter baumannii* isolates, gentamicin resistance was observed in 5/10 KTR isolates (50.0%) and 2/13 NTN isolates (15.4%), ciprofloxacin resistance in 1/2 KTR isolates (50.0%) and 2/13 NTN isolates (15.4%), and amikacin resistance in 5/10 KTR isolates (50.0%) and 3/13 NTN isolates (23.1%). Carbapenem resistance among *A. baumannii* isolates was observed more frequently in NTN isolates, with imipenem and meropenem resistance each detected in 0/10 KTR isolates (0.0%) and 3/13 NTN isolates (23.1%).

#### 2.3.2. Resistance Profiles of Gram-Positive Urinary Isolates

The antimicrobial resistance profiles of Gram-positive urinary isolates are presented in Table 4. Because the numbers of *E. faecium* and *Staphylococcus* spp. isolates were limited, comparisons involving these species should be interpreted cautiously. No ampicillin resistance was detected among *Enterococcus faecalis* isolates. In contrast, ampicillin resistance was observed in a high proportion of *E. faecium* isolates in both patient populations (2/3, 66.7% among KTRs and 12/17, 70.6% among NTN patients). Levofloxacin resistance among *E. faecalis* isolates was observed at similar frequencies among KTRs and NTN patients (17/33, 51.5% vs. 11/25, 44.0%; *p* = 0.606). Among *E. faecium* isolates, levofloxacin resistance appeared more frequent among NTN patients than among KTRs (12/15, 80.0% vs. 1/3, 33.3%), although this difference was not statistically significant (*p* = 0.172). No vancomycin resistance was detected among *Enterococcus* isolates. Linezolid resistance was observed in one *E. faecium* isolate from an NTN patient (1/15, 6.7%). Cefoxitin resistance was detected in approximately half of the *Staphylococcus* spp. isolates in both groups (8/15, 53.3% vs. 8/15, 53.3%; *p* = 1.000). No vancomycin or linezolid resistance was detected among *Staphylococcus* spp. isolates. No β-lactam resistance was observed among *Streptococcus agalactiae* isolates.

## 3. Discussion

Urinary isolate distributions and antimicrobial resistance profiles differed between kidney transplant recipients (KTRs) and non-transplant nephrology (NTN) patients in the present study. These populations may differ with respect to immunosuppression, healthcare exposure, and antimicrobial use [1,3,4,5], although direct comparisons within the same institutional setting remain limited [14,15]. However, these findings should be interpreted within the culture-based epidemiological design of the study. Because clinical symptoms, pyuria, urine culture indication, catheter status, and clinical outcomes were not systematically available, the present data cannot distinguish clinically confirmed UTI from asymptomatic bacteriuria, candiduria, colonization, or contamination. Therefore, the findings should be interpreted as differences in urinary isolate distribution and antimicrobial resistance profiles rather than as differences in clinically confirmed UTI burden.

The higher contamination rate among cultures from KTRs than among those from NTN patients was noteworthy from a pre-analytical perspective. However, because the retrospective dataset did not systematically capture urine collection method, catheter status, or adherence to clean-catch sampling instructions, the reasons underlying this difference could not be determined. Differences in specimen collection practices may influence contamination rates; however, their contribution could not be assessed in the present study [4,16,17,18]. Therefore, this finding should be interpreted cautiously and primarily as a sample-level culture outcome rather than as a patient-level clinical difference.

Compared with KTRs, NTN patients were older, with median ages of 72.0 years and 49.5 years, respectively, a pattern that may reflect differences in patient selection and clinical care pathways between transplantation and general nephrology services [1,14,19]. The KTR cohort had a higher proportion of female patients than the NTN cohort (173/220, 78.6% vs. 104/177, 58.7%) and a higher mean number of cultures with significant growth per patient (2.14 vs. 1.50). Female sex, urinary tract instrumentation, and post-transplant follow-up practices may influence the frequency of urine cultures with significant growth [2,10,16]. However, because urinary stent duration, catheter status, symptoms, pyuria, and indications for urine culture were not systematically assessed, the higher number of cultures with significant growth per patient in KTRs should not be interpreted as evidence of recurrent symptomatic UTI. Differences in culture-ordering or screening practices may also have contributed to the observed findings but could not be evaluated. These demographic and sampling-related differences should be considered when interpreting between-group comparisons of urinary isolate distribution and antimicrobial resistance profiles.

With respect to urinary isolate distribution, *E. coli* was the most frequently recovered species in both populations, consistent with previous reports [1,4,8,13]. Enterobacterales accounted for a greater proportion of isolates among KTRs than among NTN patients (77.2% vs. 41.1%), driven primarily by *E. coli* and *K. pneumoniae*. In contrast, non-fermenting Gram-negative bacteria, *Enterococcus* spp., particularly *E. faecium*, and *Candida* spp. were recovered more frequently from NTN patients. Previous studies have associated such patterns in hospitalized nephrology populations with factors such as older age, prolonged hospitalization, and urinary catheterization [1,2,16]. However, because duration of hospitalization, catheter use, and other healthcare-related exposures were not systematically assessed in the present study, their contribution to the observed distribution cannot be determined. *Candida* spp. were recovered more frequently from NTN patients than from KTRs (26.8% vs. 3.2%). However, because symptoms, pyuria, and other clinical findings were not systematically available, candiduria could not be differentiated from colonization or asymptomatic candiduria. Therefore, the higher proportion of *Candida* isolates among NTN patients should not be interpreted as evidence of a greater burden of clinically significant fungal UTI or an indication for empirical antifungal therapy [7,20]. Likewise, the lower proportion of *Candida* isolates among KTRs cannot be attributed to post-transplant nystatin prophylaxis or other clinical practices on the basis of the present data. Similarly, in the absence of clinical symptoms, pyuria, urine culture indication, and catheter-related information, *Enterococcus* spp. recovered from urine should be interpreted as urinary isolates and may represent asymptomatic bacteriuria or colonization rather than clinically confirmed enterococcal UTI [6].

Resistance patterns differed between KTRs and NTN patients, particularly among *K. pneumoniae* isolates. Among *E. coli* isolates, resistance to gentamicin and ertapenem was higher among NTN patients than among KTRs. Among *K. pneumoniae* isolates, resistance to amoxicillin–clavulanate, ceftriaxone, amikacin, gentamicin, ertapenem, and meropenem was also higher among NTN patients. In addition, ESBL production was more frequent among Enterobacterales isolates from NTN patients than among those from KTRs (55.7% vs. 41.9%, *p* = 0.013). The higher proportion of ESBL-producing Enterobacterales among NTN patients highlights the importance of local susceptibility surveillance. However, prior antimicrobial exposure, prescribing practices, and clinical outcomes were not assessed; therefore, implications for specific empirical treatment regimens cannot be determined. Although ESBL prevalence varies considerably across nephrology and transplant cohorts [16,21], the present data cannot determine whether these differences were related to prior antimicrobial exposure, duration of hospitalization, urinary tract instrumentation, or other unmeasured clinical factors.

These findings may also be interpreted within the broader context of global and transplant-specific antimicrobial resistance data. The WHO GLASS 2025 report highlighted substantial resistance among major Gram-negative bacteria, reporting that more than 40% of *E. coli* and more than 55% of *K. pneumoniae* isolates worldwide were resistant to third-generation cephalosporins [12]. In the present cohort, ceftriaxone resistance among *E. coli* isolates was 42.5% in KTRs and 54.7% in NTN patients, whereas ceftriaxone resistance among *K. pneumoniae* isolates was 43.2% and 75.0%, respectively. In a kidney transplant cohort, Korth et al. reported increasing resistance among *Klebsiella* spp. isolates to TMP–SMX, ciprofloxacin, and ceftazidime between 2009 and 2012, from 32% to 51%, 6% to 21%, and 6% to 23%, respectively, whereas resistance patterns among *E. coli* isolates remained relatively stable [5]. Although direct comparisons should be made cautiously because of differences in study design, patient populations, isolate selection, and antimicrobial susceptibility testing periods, these data support the need for population-specific local surveillance of urinary isolate resistance profiles.

Fluoroquinolone and TMP–SMX resistance frequencies were substantial in both populations. The resistance profile observed among KTRs was broadly consistent with our previous institutional KTR-only cohort, in which Gram-negative urinary isolates predominated and resistance to ciprofloxacin and TMP–SMX was high, whereas meropenem resistance remained low [13]. Although KTRs were managed according to an institutional protocol prescribing TMP–SMX prophylaxis for 12 months after transplantation, individual data on time since transplantation and active prophylactic exposure at the time of culture sampling were not systematically available. Therefore, the contribution of TMP–SMX prophylaxis to the observed resistance patterns could not be assessed at the individual level. Similarly, individual cumulative antimicrobial exposure among NTN patients was not systematically captured. Resistance patterns among aminoglycosides were agent- and species-specific. Amikacin resistance was uncommon among *E. coli* isolates in both populations and among *K. pneumoniae* isolates from KTRs; however, resistance was observed in 21.9% of *K. pneumoniae* isolates from NTN patients. These findings should not be generalized to aminoglycosides as a class. More broadly, the laboratory-based susceptibility data may support local resistance surveillance but cannot, in the absence of clinical outcome and toxicity data, establish the appropriateness of specific empirical treatment regimens.

Given the limited number of non-fermenting Gram-negative isolates, these findings should be interpreted descriptively and cautiously. Among *P. aeruginosa* isolates, resistance to imipenem, meropenem, and ceftazidime appeared more frequent among NTN patients, whereas ciprofloxacin resistance was observed in both populations. Among *A. baumannii* isolates, resistance patterns varied by antimicrobial agent: resistance to gentamicin, ciprofloxacin, and amikacin appeared more frequently among KTRs, whereas imipenem and meropenem resistance was observed only among NTN isolates. Non-fermenting Gram-negative bacteria are often associated with healthcare-related exposures [1,16]; however, prior antimicrobial use, intensive care exposure, urinary catheterization, and molecular relatedness of isolates were not assessed in the present study. Therefore, no inference can be made regarding the mechanisms underlying these resistance patterns. These low-number observations should be considered hypothesis-generating and require confirmation in larger studies.

Among Gram-positive urinary isolates, no statistically significant between-group differences in resistance rates were observed. In the absence of clinical symptoms, pyuria, and urine culture indication, *Enterococcus* isolates recovered from urine should be interpreted as urinary isolates and may represent asymptomatic bacteriuria or colonization rather than clinically confirmed enterococcal UTI [6]. However, this finding should not be interpreted as evidence of similar resistance patterns between KTRs and NTN patients, because the number of *E. faecium* isolates was particularly limited, especially among KTRs. The higher observed levofloxacin resistance among *E. faecium* isolates from NTN patients did not reach statistical significance, but the comparison was underpowered. No ampicillin resistance was detected among *E. faecalis* isolates. In contrast, ampicillin and levofloxacin resistance were observed among *E. faecium* isolates, consistent with previous reports [22,23,24]. No vancomycin resistance was detected among *Enterococcus* isolates, and no vancomycin or linezolid resistance was detected among *Staphylococcus* spp. isolates. Given the limited numbers of isolates, these findings describe the in vitro susceptibility profile of this cohort and should be interpreted cautiously. Cefoxitin resistance was detected in 53.3% of *Staphylococcus* spp. isolates in both populations; because staphylococcal species were evaluated in aggregate, this finding should not be interpreted as a measure of methicillin-resistant *S. aureus* prevalence.

This study has several limitations. First, its single-center, retrospective design limits the generalizability of the findings. The two-year study period also precluded assessment of long-term temporal trends in antimicrobial resistance. Because the analyses were primarily based on laboratory data, clinical symptoms, pyuria, indications for urine culture, catheter status, and clinical outcomes were not systematically assessed. Therefore, cultures with significant growth could not be consistently distinguished from clinically confirmed UTI, asymptomatic bacteriuria, candiduria, or colonization. Contaminated cultures were classified according to laboratory criteria, but the clinical and pre-analytical factors contributing to contamination could not be assessed. In addition, time since transplantation and active exposure to TMP–SMX or nystatin prophylaxis at the time of culture sampling were not available at the individual level. These limitations precluded adjustment for important confounders in multivariable analyses and prevented assessment of the independent effects of antimicrobial exposure on resistance patterns. Although repeated isolation of the same microorganism within a three-week interval was excluded, repeat isolates recovered after this period were retained; thus, some isolate-level observations may not have been independent, and within-patient clustering was not modeled. Furthermore, resistance profiles were assessed phenotypically, and molecular resistance mechanisms were not investigated. The limited numbers of non-fermenting Gram-negative bacteria and selected Gram-positive species also reduced the precision of some comparisons. Despite these limitations, strengths of this study include the inclusion of 397 unique patients and 1638 urine cultures over a two-year period, as well as the direct comparison of urinary isolate distributions and antimicrobial resistance profiles between KTRs and NTN patients managed at the same tertiary-care institution. This design provides local comparative surveillance data while highlighting areas that require prospective, clinically integrated investigation.

## 4. Materials and Methods

### 4.1. Study Design and Patient Population

This retrospective culture-based comparative study was conducted at Bursa Uludağ University Hospital. Urine culture results obtained from adult patients (aged ≥ 18 years) hospitalized in the Nephrology and Kidney Transplantation Units between 1 January 2024 and 31 December 2025 were reviewed. Patients were categorized as kidney transplant recipients (KTRs) or non-transplant nephrology (NTN) patients.

Culture-level analyses included all eligible urine cultures and assessed culture outcomes as significant growth, contamination, or no growth. Isolate-level analyses included urinary isolates recovered from cultures with significant growth and evaluated microorganism distribution and antimicrobial resistance profiles. Each culture with significant growth contributed a single isolate to the isolate-level analyses. Patient-level demographic analyses were restricted to unique patients with at least one urine culture showing significant growth. Repeated isolation of the same microorganism from the same patient within a three-week period was excluded; repeat isolates recovered after this interval were retained for analysis.

Demographic data, including age and sex, were retrieved from the Hospital Information Management System (MIA MED, MIA Teknoloji A.Ş., Ankara, Türkiye). Microorganism identification and antimicrobial susceptibility results were retrieved from the EpiCenter™ data management system (Becton Dickinson, Franklin Lakes, NJ, USA).

According to the institutional post-transplant prophylaxis protocol, KTRs are prescribed TMP–SMX (80/400 mg once daily) for 12 months after transplantation and oral nystatin prophylaxis during the first post-transplant month. Individual data on time since transplantation and active prophylactic antimicrobial exposure at the time of urine culture sampling were not systematically available. NTN patients admitted to the nephrology ward were not managed according to a standardized antibacterial or antifungal prophylaxis protocol.

The study protocol was approved by the Bursa Uludağ University Ethics Committee for Non-Interventional Clinical Trials (approval no. 2026/206/1-17; 8 April 2026).

### 4.2. Urine Culture Processing, Microorganism Identification, and Antimicrobial Susceptibility Testing

Urine specimens were inoculated onto Columbia agar supplemented with 5% sheep blood and eosin methylene blue (EMB) agar (BD, Heidelberg, Germany). The plates were incubated aerobically at 35–37 °C for 18–24 h. In accordance with the Clinical Microbiology Specialists Association (KLİMUD) Urine Guidelines, cultures yielding ≥ 10^5^ colony-forming units (CFU)/mL of a potential urinary pathogen were considered to show significant growth. Cultures yielding three or more distinct colony morphotypes or predominantly commensal urogenital or skin flora were classified as contaminated [25]. Contaminated cultures were included only in the sample-level culture outcome analysis shown in Table 1 and were excluded from isolate-distribution and antimicrobial-resistance analyses.

Cultures yielding three or more distinct colony morphotypes or predominantly commensal urogenital or skin flora were classified as contaminated and excluded from isolate-distribution and antimicrobial-resistance analyses.

Microorganisms from cultures with significant growth were identified by matrix-assisted laser desorption/ionization time-of-flight mass spectrometry (MALDI-TOF MS; Bruker Daltonik GmbH, Bremen, Germany). Antimicrobial susceptibility testing was performed using the BD Phoenix™ automated system (Becton Dickinson Diagnostics, Franklin Lakes, NJ, USA) and interpreted according to the European Committee on Antimicrobial Susceptibility Testing (EUCAST) clinical breakpoints (version 13.0, 2023) [26]. For binary resistance analyses, isolates categorized as resistant (R) were classified as resistant, whereas isolates categorized as susceptible, standard dosing regimen (S), or susceptible, increased exposure (I), were classified as non-resistant.

The antimicrobial agents included in the analyses varied by microorganism group. For Enterobacterales, amikacin, amoxicillin–clavulanate, ceftriaxone, ciprofloxacin, gentamicin, ertapenem, meropenem, and TMP–SMX were assessed. For *P. aeruginosa*, amikacin, ciprofloxacin, ceftazidime, imipenem, and meropenem were evaluated; for *A. baumannii*, amikacin, gentamicin, ciprofloxacin, imipenem, and meropenem were evaluated. For *Enterococcus* spp., ampicillin, levofloxacin, vancomycin, and linezolid were assessed. For *Staphylococcus* spp., cefoxitin, levofloxacin, vancomycin, and linezolid were evaluated. Resistance rates were calculated using the number of isolates tested for each antimicrobial agent as the denominator (n/N).

Internal quality control was performed using *Staphylococcus aureus* ATCC^®^ 29213™, *Escherichia coli* ATCC^®^ 25922™, and *Pseudomonas aeruginosa* ATCC^®^ 27853™.

### 4.3. Statistical Analysis

Statistical analyses were performed using IBM SPSS Statistics for Windows, version 28.0 (IBM Corp., Armonk, NY, USA). Categorical variables are presented as counts and percentages and were compared using Pearson’s chi-square test or Fisher’s exact test, as appropriate. Continuous variables are presented as mean ± standard deviation or median (minimum–maximum), as appropriate. Between-group comparisons were performed using the independent-samples *t*-test for normally distributed variables and the Mann–Whitney U test for non-normally distributed variables. Antimicrobial resistance rates were calculated using the number of isolates tested for each antimicrobial agent as the denominator. Owing to the limited number of non-fermenting Gram-negative isolates, no formal statistical comparisons of antimicrobial resistance rates were performed for these organisms, and the findings were reported descriptively. All tests were two-sided, and a *p*-value < 0.05 was considered statistically significant.

## 5. Conclusions

In this retrospective single-center culture-based study, urinary isolate distributions and in vitro antimicrobial resistance profiles differed between KTRs and NTN patients. Enterobacterales, particularly *Escherichia coli* and *Klebsiella pneumoniae*, were more frequently isolated from KTRs, whereas *Candida* spp., *Enterococcus* spp., and non-fermenting Gram-negative bacteria were more frequently recovered from NTN patients. Resistance differences were most evident among *K. pneumoniae* isolates, and ESBL production among Enterobacterales was more frequent in NTN patients. These findings should be interpreted cautiously because of the laboratory-based retrospective design and unmeasured clinical factors, including clinical symptoms, pyuria, urine culture indication, catheter status, time since transplantation, and individual antimicrobial prophylaxis exposure. Therefore, clinically confirmed UTI could not be distinguished from asymptomatic bacteriuria, candiduria, colonization, or contamination. Stratified local surveillance may help contextualize urinary isolate and susceptibility patterns across hospitalized nephrology populations. Prospective studies incorporating clinical data and outcomes are needed to clarify the determinants and clinical significance of these differences.

## Figures and Tables

**Table 1:** Sample- and patient-level characteristics of kidney transplant recipients (KTRs) and non-transplant nephrology (NTN) patients.

Characteristic	KTR	NTN	*p*-Value
**Sample-level data**	(n = 1079)	(n = 559)	
**Culture results, n (%)**			
**Significant growth with urinary isolate**	470 (43.6)	265 (47.4)	0.147
**Contamination**	173 (16.0)	25 (4.5)	<0.001
**No growth (negative)**	436 (40.4)	269 (48.1)	0.005
**Patient-level data**	(n = 220)	(n = 177)	
**Age, years, median (range)**	49.5 (21–83)	72.0 (20–98)	<0.001
**Sex, n (%)**			<0.001
**Female**	173 (78.6)	104 (58.7)	
**Male**	47 (21.4)	73 (41.3)	

KTR, kidney transplant recipients; NTN, non-transplant nephrology patients.

**Table 2:** Distribution of urinary isolates in KTRs and NTN patients.

Microorganism	KTR (n = 470)	NTN (n = 265)	*p*-Value
**Enterobacterales, total**	363 (77.2)	109 (41.1)	<0.001
***Escherichia coli***	248 (52.8)	64 (24.2)	<0.001
***Klebsiella pneumoniae***	95 (20.2)	32 (12.1)	0.007
***Proteus mirabilis***	12 (2.6)	3 (1.1)	0.300
**Other Enterobacterales ^a^**	8 (1.7)	10 (3.8)	0.130
**Non-fermenting Gram-negative bacteria, total**	16 (3.4)	25 (9.4)	0.001
***Acinetobacter baumannii***	10 (2.1)	13 (4.9)	0.063
***Pseudomonas aeruginosa***	6 (1.3)	5 (1.9)	0.740
**Other non-fermenting Gram-negative bacteria ^b^**	0 (0.0)	7 (2.6)	0.002
***Enterococcus* spp., total**	36 (7.7)	42 (15.8)	<0.001
***Enterococcus faecalis***	33 (7.0)	25 (9.4)	0.310
***Enterococcus faecium***	3 (0.6)	17 (6.4)	<0.001
***Staphylococcus* spp., total**	16 (3.4)	15 (5.7)	0.180
***Staphylococcus aureus***	1 (0.2)	5 (1.9)	0.046
**Other *Staphylococcus* spp. ^c^**	15 (3.2)	10 (3.8)	0.840
***Streptococcus agalactiae***	14 (3.0)	2 (0.8)	0.085
***Candida* spp., total**	15 (3.2)	71 (26.8)	<0.001
***Candida albicans***	3 (0.6)	35 (13.2)	<0.001
***Candida glabrata***	1 (0.2)	12 (4.5)	<0.001
**Other *Candida* spp. ^d^**	11 (2.3)	24 (9.1)	<0.001
**Other microorganisms**	10 (2.1)	1 (0.4)	—

KTR, kidney transplant recipients; NTN, non-transplant nephrology patients. ^a^: *Enterobacter* species, *Citrobacter freundii*, *Morganella morganii*, *Serratia marcescens*. ^b^: *Stenotrophomonas maltophilia*, *Burkholderia cepacia complex*. ^c^: *Staphylococcus haemolyticus*, *Staphylococcus lugdunensis*, *Staphylococcus epidermidis*. ^d^: *Candida kefyr*, *Candida krusei*, *Candida tropicalis*, *Candida lusitaniae*, *Candida parapsilosis*, *Candida dubliniensis*.

**Table 3:** Antimicrobial resistance profiles of *Escherichia coli* and *Klebsiella pneumoniae* urinary isolates from KTRs and NTN patients.

Bacterial Species/Antimicrobial Agent	KTR, n/N (%)	NTN, n/N (%)	*p*-Value
***Escherichia coli***			
**Amikacin**	2/248 (0.8)	1/64 (1.6)	0.499
**Amoxicillin–clavulanate**	67/127 (52.8)	17/29 (58.6)	0.568
**Ceftriaxone**	105/247 (42.5)	35/64 (54.7)	0.081
**Ciprofloxacin**	118/224 (52.7)	36/61 (59.0)	0.379
**Gentamicin**	34/248 (13.7)	18/64 (28.1)	0.006
**Ertapenem**	15/248 (6.0)	12/64 (18.8)	0.001
**Meropenem**	3/244 (1.2)	2/63 (3.2)	0.273
**TMP–SMX**	109/248 (44.0)	27/64 (42.2)	0.800
***Klebsiella pneumoniae***			
**Amikacin**	1/95 (1.1)	7/32 (21.9)	<0.001
**Amoxicillin–clavulanate**	30/55 (54.5)	17/19 (89.5)	0.006
**Ceftriaxone**	41/95 (43.2)	24/32 (75.0)	0.002
**Ciprofloxacin**	40/82 (48.8)	19/30 (63.3)	0.172
**Gentamicin**	5/95 (5.3)	10/32 (31.3)	<0.001
**Ertapenem**	18/95 (18.9)	12/32 (37.5)	0.033
**Meropenem**	2/93 (2.2)	8/32 (25.0)	<0.001
**TMP–SMX**	52/95 (54.7)	20/32 (62.5)	0.443

n, number of resistant isolates; N, number of isolates tested for the respective antimicrobial agent. Comparisons of resistance proportions between KTRs and NTN patients were performed using Pearson’s chi-square test when all expected cell counts were ≥5 and Fisher’s exact test otherwise. KTR, kidney transplant recipients; NTN, non-transplant nephrology patients; TMP–SMX, trimethoprim–sulfamethoxazole.

**Table 4:** Antimicrobial resistance profiles of Gram-positive urinary isolates from KTRs and NTN patients.

Microorganism/Antimicrobial Agent	KTR, n/N (%)	NTN, n/N (%)	*p*-Value
***Enterococcus faecalis***			
**Ampicillin**	0/33 (0.0)	0/25 (0.0)	–
**Vancomycin**	0/33 (0.0)	0/25 (0.0)	–
**Linezolid**	0/33 (0.0)	0/25 (0.0)	–
**Levofloxacin**	17/33 (51.5)	11/25 (44.0)	0.606
***Enterococcus faecium***			
**Ampicillin**	2/3 (66.7)	12/17 (70.6)	1.000
**Vancomycin**	0/3 (0.0)	0/16 (0.0)	–
**Linezolid**	0/3 (0.0)	1/15 (6.7)	1.000
**Levofloxacin**	1/3 (33.3)	12/15 (80.0)	0.172
***Staphylococcus* spp.**			
**Cefoxitin**	8/15 (53.3)	8/15 (53.3)	1.000
**Vancomycin**	0/16 (0.0)	0/15 (0.0)	–
**Linezolid**	0/16 (0.0)	0/16 (0.0)	–
**Levofloxacin**	6/16 (37.5)	3/16 (18.8)	0.433

n, number of resistant isolates; N, number of isolates tested for the respective antimicrobial agent. –, not calculated because no resistant isolates were detected in either group. KTR, kidney transplant recipients; NTN, non-transplant nephrology patients.

## Data Availability

Anonymized data are available on request.

## Abbreviations

The following abbreviations are used in this manuscript:

CFU	Colony-forming units
CoNS	Coagulase-negative staphylococci
ESBL	Extended-spectrum β-lactamase
EUCAST	European Committee on Antimicrobial Susceptibility Testing
KTR	Kidney transplant recipient
MALDI–TOF MS	Matrix-assisted laser desorption/ionization time-of-flight mass spectrometry
MDR	Multidrug resistance
NTN	Non-transplant nephrology
TMP–SMX	Trimethoprim–sulfamethoxazole
UTI	Urinary tract infection.

## References

[1] Mir S.S., Ali E., Alghamdi S.A.A., Alghamdi N.M., Alharbi R.A., Sindi A.A.A., Zaeri A.A. (2025). Antimicrobial Resistance in Urinary Tract Infections Among Patients with and Without Renal Comorbidities: A Retrospective Study from Al-Baha, Saudi Arabia. Pathogens.

[2] Elsayed M.M., Elzayat D.M., Mekawy R.M., Mahdy S.M., Imam S.T., El-Hassan H.A.A., Elwaraky R.I., Emam E.M., Gawad M.A. (2025). Urinary Tract Infection in Patients with Chronic Kidney Disease: A Retrospective Observational Study at Outpatient Nephrology Clinics. J. Egypt. Soc. Nephrol. Transplant..

[3] Pinchera B., Carrano R., Schettino E., D’Agostino A., Trucillo E., Cuccurullo F., Salemi F., Piccione A., Gentile I. (2025). Urinary Tract Infections in Kidney Transplant Patients Admitted to Hospital: A Real-Life Experience. World J. Transplant..

[4] Goldman J.D., Julian K. (2019). Urinary Tract Infections in Solid Organ Transplant Recipients: Guidelines from the American Society of Transplantation Infectious Diseases Community of Practice. Clin. Transplant..

[5] Korth J., Kukalla J., Rath P.M., Dolff S., Krull M., Guberina H., Bienholz A., Wilde B., Becker S., Ross B. (2017). Increased Resistance of Gram-Negative Urinary Pathogens after Kidney Transplantation. BMC Nephrol..

[6] Nicolle L.E., Gupta K., Bradley S.F., Colgan R., DeMuri G.P., Drekonja D., Eckert L.O., Geerlings S.E., Köves B., Hooton T.M. (2019). Clinical Practice Guideline for the Management of Asymptomatic Bacteriuria: 2019 Update by the Infectious Diseases Society of America. Clin. Infect. Dis..

[7] Pappas P.G., Kauffman C.A., Andes D.R., Clancy C.J., Marr K.A., Ostrosky-Zeichner L., Reboli A.C., Schuster M.G., Vazquez J.A., Walsh T.J. (2016). Clinical Practice Guideline for the Management of Candidiasis: 2016 Update by the Infectious Diseases Society of America. Clin. Infect. Dis..

[8] Mareș C., Petca R.C., Popescu R.I., Petca A., Mulțescu R., Bulai C.A., Ene C.V., Geavlete P.A., Geavlete B.F., Jinga V. (2024). Update on Urinary Tract Infection Antibiotic Resistance: A Retrospective Study in Females in Conjunction with Clinical Data. Life.

[9] Brizendine K.D., Richter S.S., Cober E.D., van Duin D. (2015). Carbapenem-Resistant *Klebsiella pneumoniae* Urinary Tract Infection Following Solid Organ Transplantation. Antimicrob. Agents Chemother..

[10] Bodro M., Sanclemente G., Lipperheide I., Allali M., Marco F., Bosch J., Cofan F., Ricart M.J., Esforzado N., Oppenheimer F. (2015). Impact of Urinary Tract Infections on Short-Term Kidney Graft Outcome. Clin. Microbiol. Infect..

[11] World Health Organization Antimicrobial Resistance. https://www.who.int/news-room/fact-sheets/detail/antimicrobial-resistance.

[12] World Health Organization (2025). Global Antibiotic Resistance Surveillance Report 2025.

[13] Çalışır B., Çalışır A.İ., Rodoplu O., Yıldız A., Ersoy A., Özakın C. (2025). Antibiotic Resistance in Urinary Pathogens Among Kidney Transplant Recipients: A Persistent Threat. Antibiotics.

[14] Gerges-Knafl D., Pichler P., Zimprich A., Hotzy C., Barousch W., Lang R.M., Lobmeyr E., Baumgartner-Parzer S., Wagner L., Winnicki W. (2020). The Urinary Microbiome Shows Different Bacterial Genera in Renal Transplant Recipients and Non-Transplant Patients at Time of Acute Kidney Injury: A Pilot Study. BMC Nephrol..

[15] Jaworska M.M., Pecyna P., Jaskiewicz K., Rydzanicz M., Kaluzna M., Pawlaczyk K., Ploski R., Nowak-Malczewska D.M., Karolak J.A., Gajecka M. (2023). Differences in the Composition of the Bacterial Element of the Urinary Tract Microbiome in Patients Undergoing Dialysis and Patients after Kidney Transplantation. Front. Microbiol..

[16] Michno M., Sydor A., Wałaszek M., Sułowicz W. (2018). Microbiology and Drug Resistance of Pathogens in Patients Hospitalized at the Nephrology Department in the South of Poland. Pol. J. Microbiol..

[17] LaRocco M.T., Franek J., Leibach E.K., Weissfeld A.S., Kraft C.S., Sautter R.L., Baselski V., Rodahl D., Peterson E.J., Cornish N.E. (2016). Effectiveness of Preanalytic Practices on Contamination and Diagnostic Accuracy of Urine Cultures: A Laboratory Medicine Best Practices Systematic Review and Meta-Analysis. Clin. Microbiol. Rev..

[18] Thompson E.R., Hosgood S.A., Nicholson M.L., Wilson C.H. (2018). Early versus Late Ureteric Stent Removal after Kidney Transplantation. Cochrane Database Syst. Rev..

[19] Coutinho G.M.M., Silva E.C.D., Campanharo C.R.V., Belasco A.G.S., Fonseca C.D.D., Barbosa D.A. (2021). Urinary Tract Infection in Patients with Chronic Kidney Disease under Conservative Treatment. Rev. Bras. Enferm..

[20] Chotiprasitsakul D., Kijnithikul A., Uamkhayan A., Santanirand P. (2021). Predictive Value of Urinalysis and Recent Antibiotic Exposure to Distinguish Between Bacteriuria, Candiduria, and No-Growth Urine. Infect. Drug Resist..

[21] Velioglu A., Guneri G., Arikan H., Asicioglu E., Tigen E.T., Tanidir Y., Tinay İ., Yegen C., Tuglular S. (2021). Incidence and Risk Factors for Urinary Tract Infections in the First Year after Renal Transplantation. PLoS ONE.

[22] Gong L., Zhang L., Liu X., Odilov B., Li S., Hu Z., Xiao X. (2021). Distribution and Antibiotic Susceptibility Pattern of Multidrug-Resistant Bacteria and Risk Factors among Kidney Transplantation Recipients with Infections over 13 Years: A Retrospective Study. Infect. Drug Resist..

[23] Haque Sumon A.H.M.S., Al-Mahmood M.R., Islam K.A., Karim A.N.M.E., Aker P., Ullah A., Rashid M.A., Hasan M.N. (2023). Multidrug Resistance Urinary Tract Infection in Chronic Kidney Disease Patients: An Observational Study. Cureus.

[24] Luo Q., Lu P., Chen Y., Shen P., Zheng B., Ji J., Ying C., Liu Z., Xiao Y. (2024). ESKAPE in China: Epidemiology and Characteristics of Antibiotic Resistance. Emerg. Microbes Infect..

[25] Ministry of Health of the Republic of Türkiye, KLİMUD (2019). Antibiogram Interpretation Criteria and Restricted Reporting Rules: National Microbiology Standards.

[26] The European Committee on Antimicrobial Susceptibility Testing (2023). Breakpoint Tables for Interpretation of MICs and Zone Diameters, Version 13.0.

